# An Unusual Presentation of Costal Intraspinal Osteochondroma Causing Compressive Myelopathy in a Patient With Hereditary Multiple Exostoses: A Case Report and Literature Review

**DOI:** 10.7759/cureus.71036

**Published:** 2024-10-07

**Authors:** Jerin Jeevo, Ankith N V, Mahesh Shekoba

**Affiliations:** 1 Department of Orthopaedics, St. John's Medical College and Hospital, Bangalore, IND

**Keywords:** compressive myelopathy, costal intraspinal osteochondroma, en block excision, hereditary multiple exostosis, spinal exostosis

## Abstract

A 22-year-old man presented to us with back pain for four months, inability to walk, and weakness in both lower limbs. Clinical examination revealed multiple swellings in the body, motor weakness, paresthesia, and upper motor neuron signs. Both magnetic resonance imaging (MRI) and computed tomography (CT) revealed an abnormal bone mass protruding into the spinal canal from the posterior aspect of the ninth rib through the D9-D10 neural foramen. After routine preoperative evaluation, the lesion was removed en masse along with the rib to which it was attached. Osteochondroma was the histological diagnosis. After the procedure, the patient’s symptoms subsided, and he could return to his daily activities. From this case, we recommend performing whole spine MRI screening for all patients with hereditary multiple exostoses to diagnose intraspinal lesions that need to be addressed early to have a good postoperative outcome.

## Introduction

Hereditary multiple exostoses (HME) is a condition characterized by excessive growths of multiple osteochondromas, which are cartilage-capped benign bone tumors that grow away from the metaphysis of long bones. Osteochondromas can be related to deformities of bone, short stature, early osteoarthritis, compressive neuropathies, restricted skeletal growth, and joint motion. Almost all the individuals who are affected are diagnosed by the age of 12 [1]. The transmission of disease is autosomal-dominant. The mutations of the *EXT1* and *EXT2* genes have been found, respectively, in 28-65% and 21-61% of the affected patients [2]. Up until 2007, there have only been 51 documented occurrences of solitary vertebral osteochondromas with spinal cord compression. Compared to isolated osteochondromas, the frequency of spinal involvement (3% of cases) and neurological sequelae is higher in HME [3].

## Case presentation

**Figure 1:**
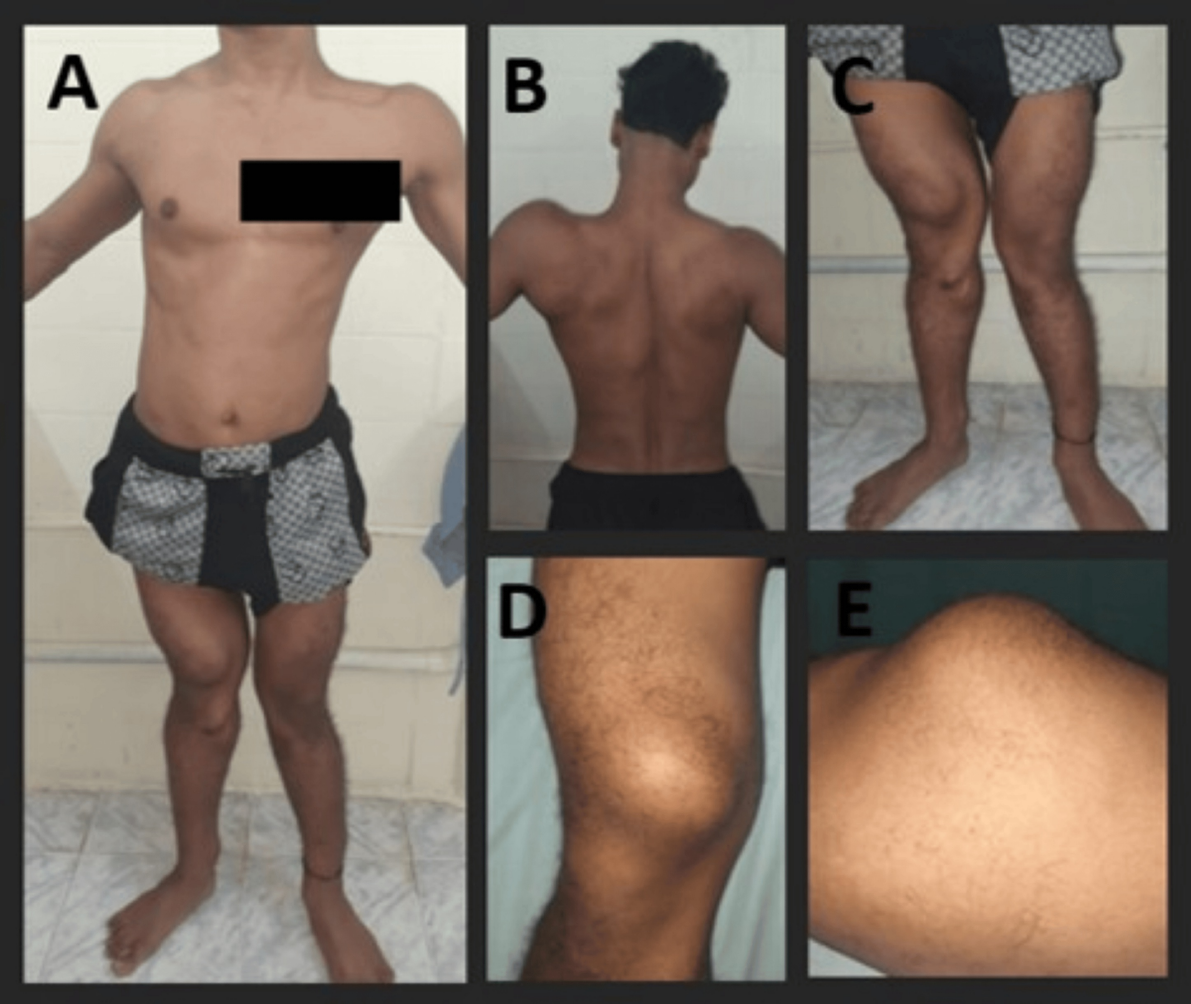
Clinical image of the patient. (A) Multiple bony swellings in the body. (B) Bony swellings in the left scapula. (C) Valgus deformity of the knee. (D and E) Bony swelling in the right distal thigh.

We present a case of a 22-year-old man who presented with complaints of multiple swellings in his body, inability to walk, and weakness in both lower limbs for four months. The weakness was acute in onset, gradually progressive, and ultimately left him bedridden after four months. He also underwent surgery for the excision of a bony mass in his left knee six months ago. On examination, he had bony swellings over his right scapula and both knees, specifically on the medial femoral condyle, causing valgus deformity (Figure 1A-E).

**Figure 2:**
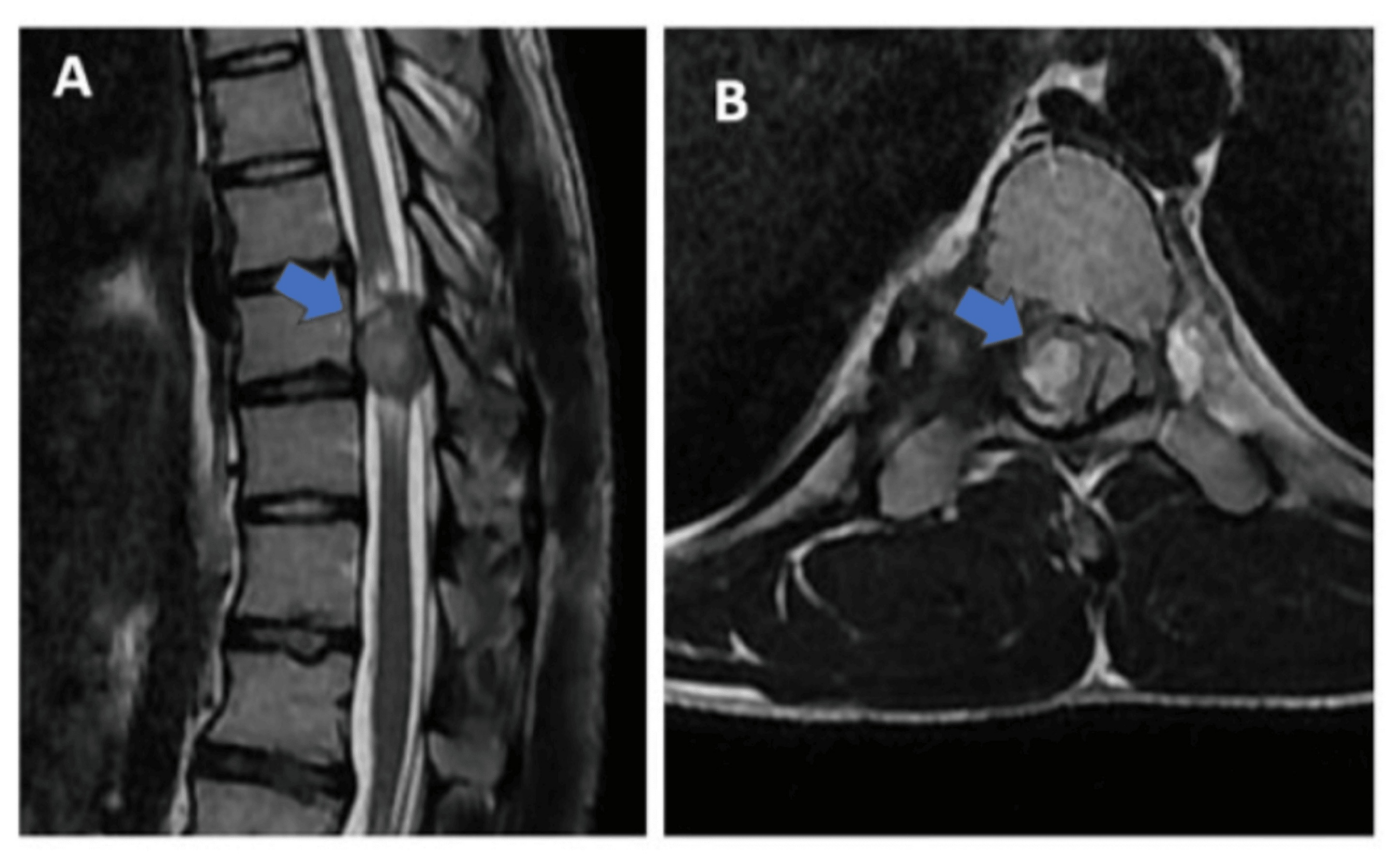
MRI spine showing hypointense lesion in the spinal canal. (A) Blue arrow showing the lesion in the spinal canal with cord edema. (B) Blue arrow showing the lesion arising from the posterior aspect of the ninth rib extending into the D9-D10 neural foramen, compressing the exiting nerve root and displacing the spinal cord towards the left side, causing compression. MRI: magnetic resonance imaging.

**Figure 3:**
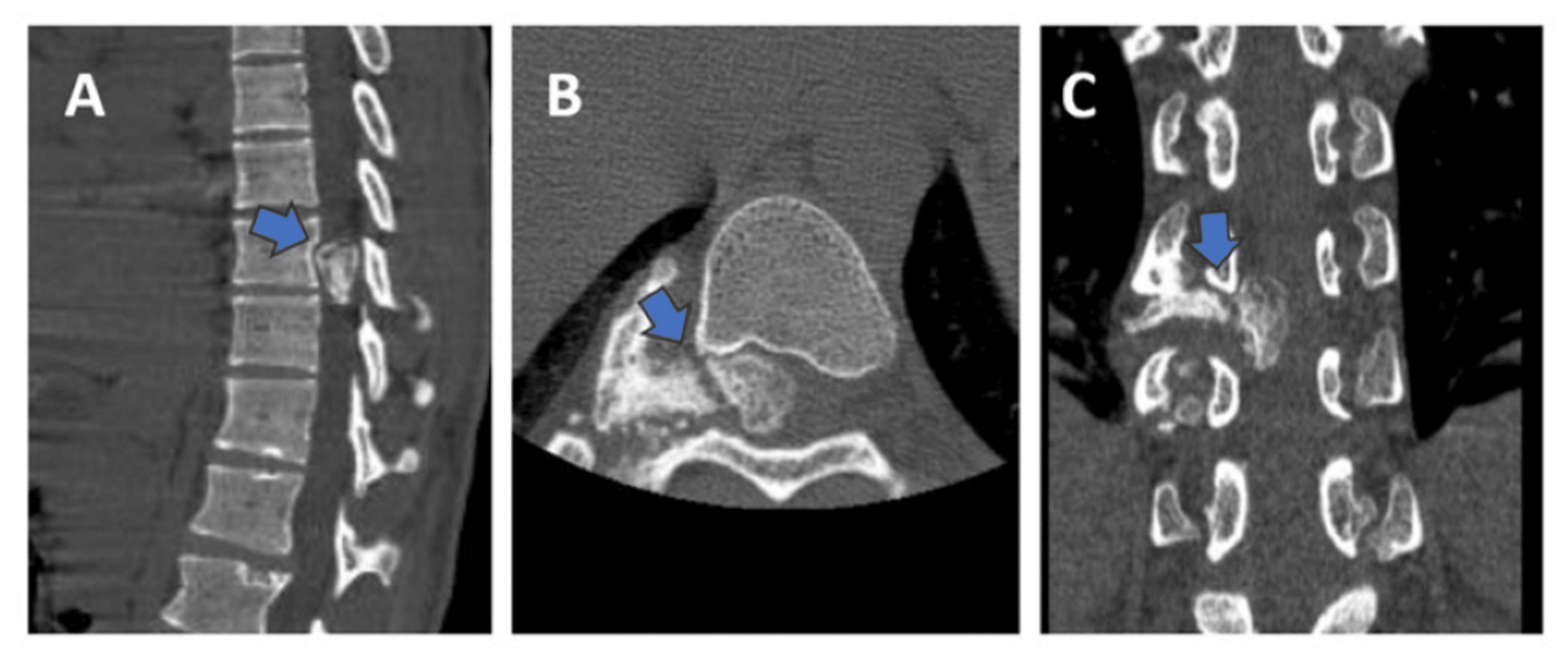
CT spine showing a bony mass arising from the posterior aspect of the ninth rib head extending into the D9-D10 neural foramen. (A) Sagittal section of CT spine showing a bony mass in the spinal canal and posterior scalloping of the D10 vertebra. (B and C) Axial and coronal sections of CT spine showing a bony mass arising from the posterior aspect of the ninth rib head extending into the D9-D10 neural foramen. CT: computed tomography.

Neurological examination revealed hypertonia in both lower limbs. Power in both lower limbs was 2/5. Deep tendon reflexes were exaggerated. Both patella and ankle clonus were positive. The Babinski sign was positive. Anteroposterior (AP) and lateral radiographs of the spine were taken and showed no obvious findings. Whole spine magnetic resonance imaging (MRI) was taken, which showed a hypointense lesion displacing the spinal cord towards the left causing compression and cord edema (Figure 2A, B). A computed tomography (CT) scan of the whole spine showed a bony mass arising from the posterior aspect of the ninth rib, entering the spinal canal through D9-D10 neural foramen and causing posterior scalloping of the D10 vertebra (Figure 3A-C).

**Figure 4:**
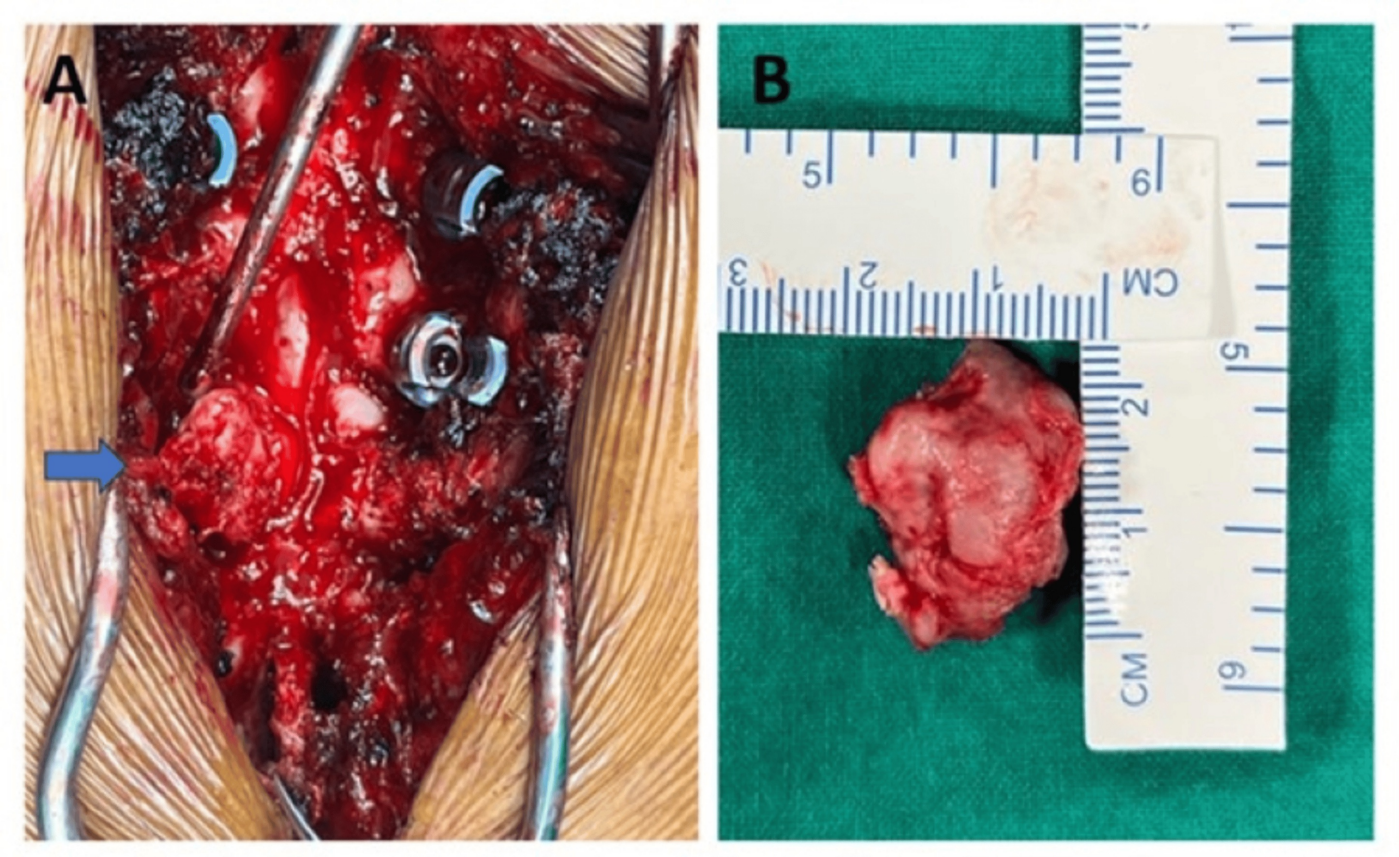
Intraoperative images. (A) Blue arrow indicating the lesion compressing the thecal sac, with one of the roots wrapped over it. (B) Dimensions of the lesion, approximately 2 x 2 cm.

The patient underwent a routine preoperative evaluation and was taken up for decompression surgery. Through a conventional posterior midline approach D9, D10 laminectomy was done. Facetectomy and transverse process excision were carried out on the right side. The lesion was found to be attached to the D9 rib. The right D9 root was wrapped around the osteochondroma mass (Figure 4A, B).

**Figure 5:**
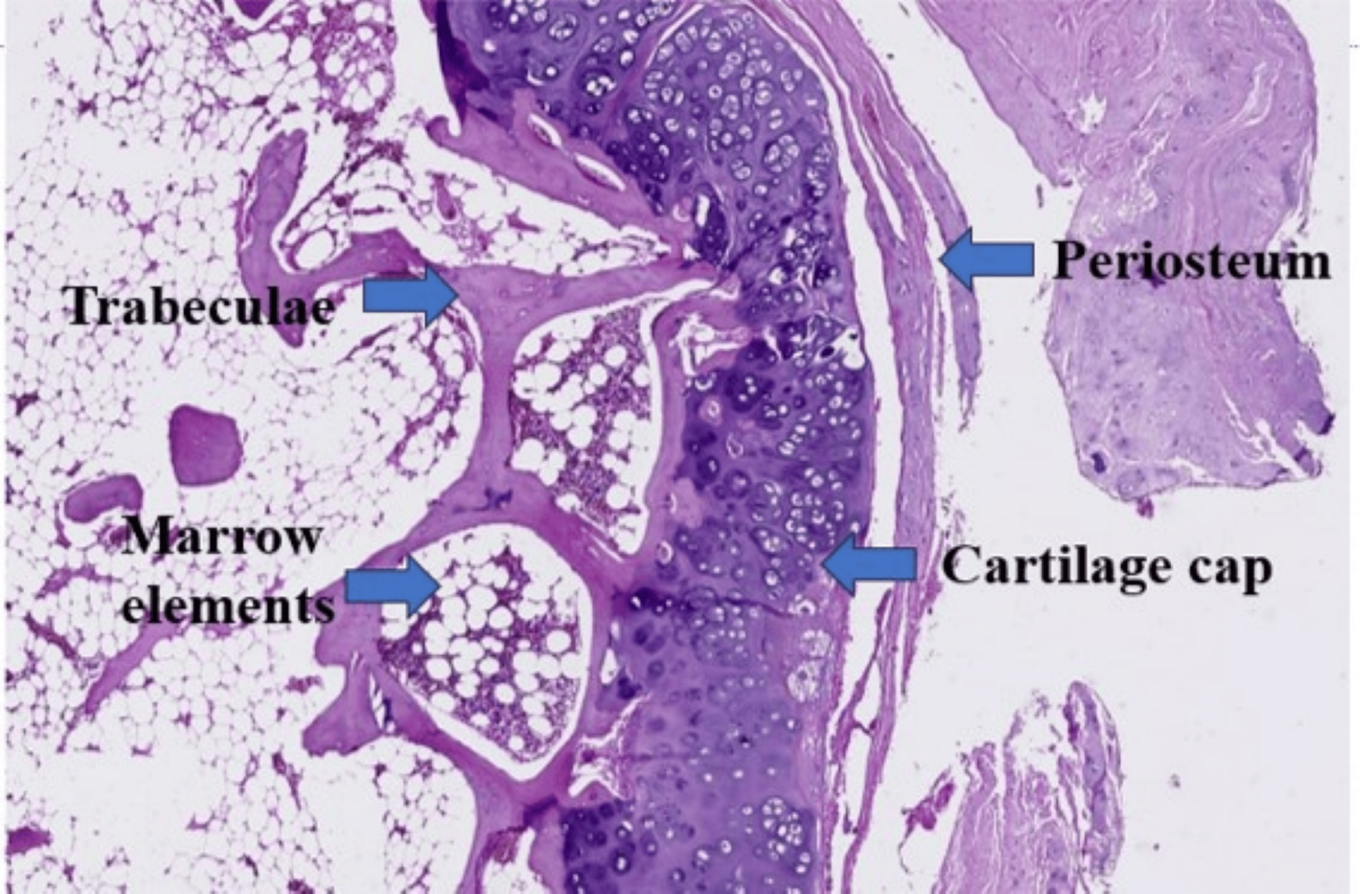
Histopathology image.

Gentle retraction of the root was done, and the lesion was removed en masse along with the part of the rib to which it was attached. Instrumentation with pedicular screws was done from D7 to D11. The lesion was sent for histopathological analysis (Figure 5).

**Figure 6:**
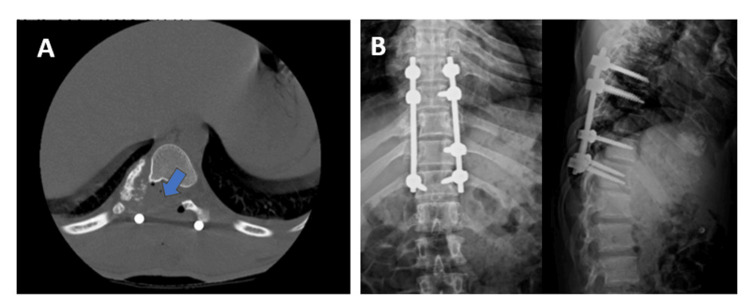
Postoperative CT and radiographs. (A) CT scan: Blue arrow showing the absence of lesion in the spinal canal. (B) AP and lateral spine radiographs: To confirm the position of the pedicular screws. AP: anteroposterior; CT: computed tomography.

Postoperative CT was performed to confirm the adequacy of decompression in the affected level. Radiographs were also taken to confirm the positions of the pedicular screws (Figure 6A, B).

After suture removal, the patient was asked to follow up at six weeks, three months, and one year. At six weeks of postoperative follow-up, the patient was able to sit without support. At three months of postoperative follow-up, the patient was able to walk without support. At one year of postoperative follow-up, the patient’s symptoms had subsided completely, and he was able to perform his daily activities and occupation.

## Discussion

Generally, patients present with a palpable mass around the joints. The most frequently affected joints are the knees, shoulders, ankles, and wrists [4]. Involvement of the proximal femur can lead to valgus deformity of the hip, while involvement of the acetabulum can result in femoral acetabular dysplasia, subsequently leading to osteoarthritis [5].

The most prevalent abnormality observed in cases of knee joint involvement is genu valgum, usually caused by proximal tibial angulation. Occasionally, an oblique joint line can be formed due to the affection of the distal femur [6]. Ankle joint deformation is also associated with valgus deformity. The oblique growth plate and joint line that result from a shorter distal fibula than distal tibia usually cause the talus to shift medially [7].

In the past, spinal exostosis was neglected. However, it is estimated that 68% of the patients could have spinal osteochondromas. Studies that link scoliosis to HME are rare [8]. Since most patients are asymptomatic at birth, genetic screening of the offspring of affected families is the only method of diagnosis at birth [9]. 

In most cases, radiographs can be used to evaluate the location and morphology of osteochondromas. They are also beneficial in detecting the HME-related deformities. The distal femur "Erlenmeyer flask" deformity, pseudo-Madelung deformity, and ulnar shortening were linked to around 33% of the individuals with HME. To assess the anatomical relationship between neighboring structures, imaging using MRI or CT scan is typically required. Bursitis resulting from aberrant mechanical stress surrounding the lesion can be confirmed by MRI [10].

Measuring the cartilage cap is one of the methods to anticipate the malignant conversion of the lesion. In adults, when the thickness of the cartilage cap is more than 2 cm, malignant transformation should be suspected. The presence of irregular calcifications, lytic lesion inside the exostosis, and soft tissue mass surrounding the exostosis are other signs to predict malignant transformation [11].

According to a study by Roach et al., patients whose spines were screened with MRI showed 68% spinal exostosis involvement. Of which, 27% had spinal exostosis that was impinging their spinal cord, which was higher than expected [8]. Since most of the previous research were dependent on X-ray screening, most of the patients with spinal exostosis were missed. According to other authors, 15% of the patients having spinal exostosis were having myelopathy. Most of the patients with HME who experienced minor trauma showed signs of acute neurological syndromes. Therefore, the authors recommend screening the spine by MRI/CT for all patients with HME [9].

There are very few case reports about osteochondroma arising from a rib causing compressive myelopathy. But there are many case reports about osteochondroma arising from the posterior elements of the spine (Table 1).

**Table 1:** Different studies with various origin of exostosis, duration of neurological symptoms, and postoperative outcome.

Study	Origin of exostosis	Duration of neurological symptoms	Postoperative outcome
Mehrian et al [3]	D9 lamina	One year	Good
Okuyama et al [12]	D11 inferior articular process	Five years	Poor
Khosla et al. [13]	C1 lamina	Two years	Good
Upadhyaya et al. [14]	D4 right pedicle	Five months	Good
Al Kaissi et al. [15]	D3-5 pedicle	Not reported	Good
Calvo et al. [16]	D3 pedicle	Two months	Good
Our study	Ninth rib extending into D9 neural foramen	Four months	Good

## Conclusions

Apart from common factors such as trauma, discopathy, and metastases, uncommon entities like osteochondroma should also be considered in situations of spinal cord compression. This is especially crucial for individuals with HME. Based on this case report, we recommend that whole spine MRI screening should be performed for all patients with HME to diagnose intraspinal lesions. If intraspinal lesions are diagnosed, those patients should be kept under regular follow-up so that any neurological symptom or sign when identified can be intervened at the earliest to ensure a favorable outcome following surgery.
